# **γδ** T cells and cancer

**DOI:** 10.1172/JCI209159

**Published:** 2026-09-01

**Authors:** Lukas Bolini, Seth B. Coffelt, Bruno Silva-Santos, David L. Wiest, Lorenzo Galluzzi

**Affiliations:** 1 Cancer Signaling and Microenvironment Program, Fox Chase Cancer Center, Philadelphia, Pennsylvania, USA.; 2 Cancer Research UK Scotland Institute, Glasgow, United Kingdom.; 3 School of Cancer Sciences, University of Glasgow, Glasgow, United Kingdom.; 4 Faculdade de Medicina, Universidade de Lisboa, Lisbon, Portugal.; 5 Gulbenkian Institute for Molecular Medicine, Lisbon, Portugal.; 6 Nuclear Dynamics and Cancer Program, Fox Chase Cancer Center, Philadelphia, Pennsylvania, USA.

## Abstract

γδ T cells are a subset of lymphoid cells that, unlike their αβ lineage counterparts, express a heterodimeric TCR that mostly operates in an MHC-independent manner. γδ T cells are abundant in barrier tissues, where they continuously monitor epithelial cells for signs of stress or damage. Thus, γδ T cells are among the first responders to pathophysiological conditions, including viral infection and oncogenesis. Human γδ T cells can be classified based on TCR γ and δ chain usage into three main subsets: (a) Vγ9^+^Vδ2^+^ cells, accounting for most circulating γδ T cells; (b) Vδ1^+^ cells, which are common in epithelial linings, and (c) Vδ3^+^ T cells, which are fairly rare but exhibit unique specificities. Moreover, both human and murine γδ T cells can assume a spectrum of states with divergent phenotypic and functional properties. Accumulating evidence demonstrates that γδ T cells can mediate robust anticancer effects or support tumor progression and resistance to therapy, depending on numerous variables, including functional state and tumor type. Here, we critically discuss the context-dependent interaction between γδ T cells and cancer, focusing on recent developments and the challenges facing current efforts to manipulate this versatile lymphocyte subset for therapeutic purposes.

## Introduction

**Figure 1:**
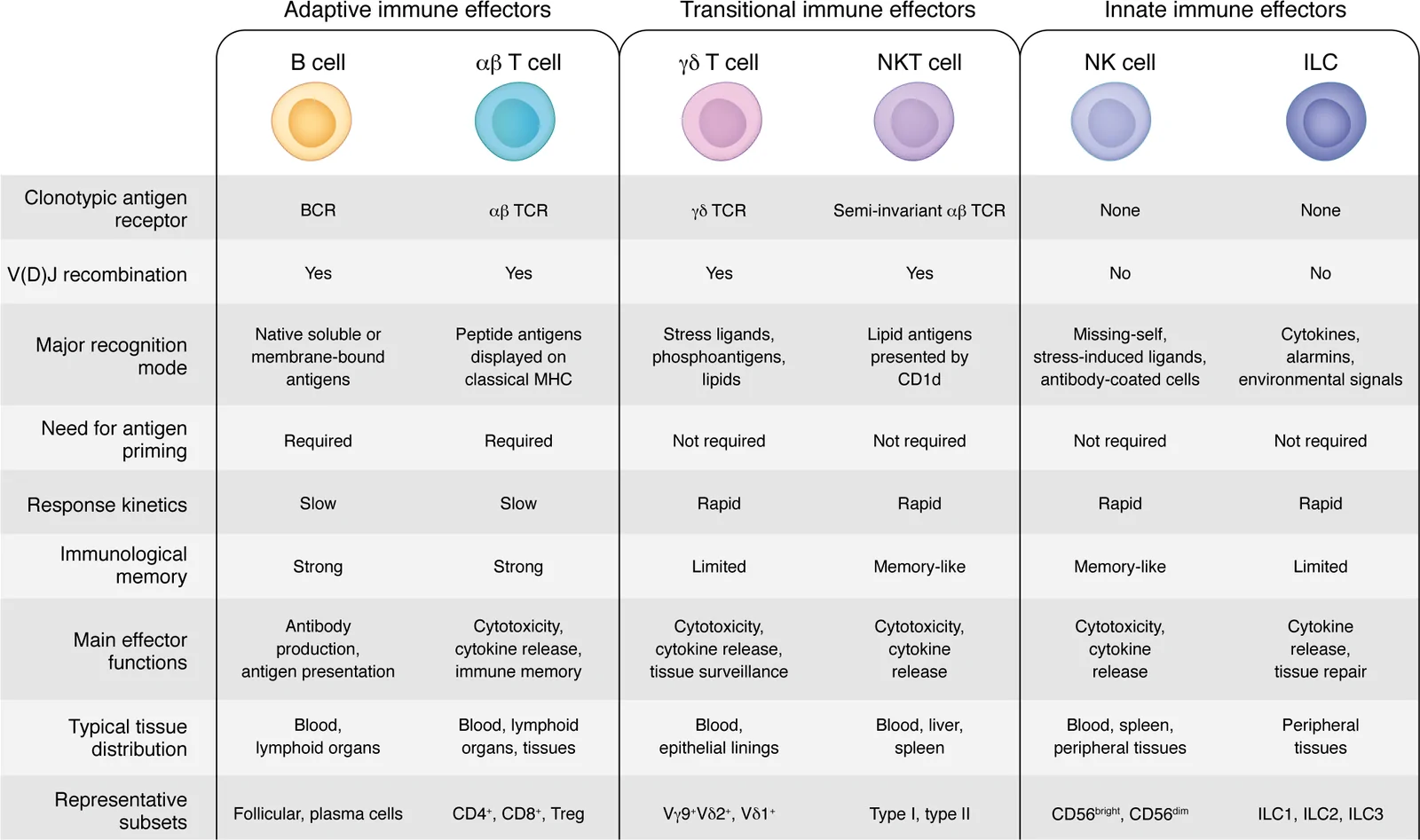
Key features of major lymphoid cell compartments in mammals. Mammalian lymphoid cells encompass populations with an innate behavior, rapidly responding to nonspecific signal of cellular stress irrespective of antigen presentation on MHC class I molecules, such as NK cells and other innate lymphoid cell (ILC) subsets. Moreover, the mammalian immune system relies on various populations of cells mounting exquisite antigen-specific, adaptive responses associated with molecular memory, like conventional αδ T cells and B lymphocytes. Finally, multiple mammalian immune cell populations have evolved to exhibit phenotypes and/or functional traits at the interface between innate and adaptive immunity, including γδ and NK T cells. Each of these populations encompasses a spectrum of phenotypically and functionally distinct subsets that mediate highly specialized effector functions, including cellular cytotoxicity, proinflammatory or immunosuppressive cytokine secretion, antibody production, and antigen presentation. BCR, B cell receptor; TCR, T cell receptor.

γδ T cells are lymphocytes operating at the interface between innate and adaptive immunity (1, 2). Indeed, γδ T cells share several properties with NK cells and other innate lymphoid cells (ILCs) (3–5) in that both γδ T cells and ILCs are activated rapidly and in the absence of priming by immune cells like DCs (1). Moreover, γδ T cells and ILCs recognize stress-induced molecules independent of MHC complexes (3, 4, 6, 7). Conversely, γδ T cells resemble conventional T cells as they arise from hematopoietic precursors in the thymus and express a somatically rearranged heterodimeric TCR associated with CD3 signaling subunits (3). However, unlike conventional T cells (carrying an αβ TCR), which recognize peptide antigens presented on MHC class I (CD8^+^ T cells) or II (CD4^+^ T cells) molecules (3), γδ T cells (generally CD4^–^CD8^–^) recognize nonpeptide ligands, metabolites, or stress-induced surface proteins in their native state and independent of MHC presentation (3, 7, 8) (Figure 1).

γδ T cells are critical for the preservation of tissue homeostasis and early responses to viral infection (9, 10). Indeed, through the γδ TCR and other receptors shared with ILCs, γδ T cells recognize molecules induced by metabolic dysregulation, cellular damage, or infection, enabling them to detect infected or transformed cells rapidly and initiate a response before the engagement of conventional adaptive immunity (1, 10). Activated γδ T cells mediate secretory functions, releasing a context-dependent spectrum of cytokines that encompass IFN-γ, TNF, and IL17A (3), as well as cytotoxic effects, via perforin 1–dependent (PRF1-dependent) and granzyme B–dependent (GZMB-dependent) mechanisms or by triggering FAS signaling (3, 11). Finally, some γδ T cell subsets (notably, human Vγ9^+^Vδ2^+^ T cells) present peptide antigens to αβ T cells together with adequate costimulatory signals, contributing to αβ T cell priming (12, 13).

Substantial differences exist between human and mouse γδ T cells in terms of subset composition, tissue distribution, and TCR specificity (3), implying that considerable caution must be employed when translating findings from one organism to the other. Most human circulating γδ T cells express a Vγ9^+^Vδ2^+^ TCR that detects phosphorylated metabolites, including isopentenyl pyrophosphate (IPP) and E-4-hydroxy-3-methyl-but-2-enyl pyrophosphate (HMBPP) (14–17). This depends on BTN3A1/BTN2A1 dimers that undergo conformational alterations and are exposed on the target cell surface upon binding to IPP or HMBPP intracellularly (18–22). A second major human γδ T cell subset expresses Vδ1 TCR chains and predominates in epithelial linings (and epithelial neoplasms) (23). Human Vδ3^+^ T cells are rare but (to some extent) enriched in the liver and intestine (23). Finally, while some minor Vδ1^+^ and Vδ3^+^ T cell subsets recognize lipids bound to CD1d (resembling NK T cells) (24, 25), they also respond to stress signals commonly recognized by ILCs and to noncanonical MHC molecules (26–28).

Mice lack an equivalent of human Vγ9^+^Vδ2^+^ T cells and do not express functional BTN3A1 and BTN2A1 homologs, but they have various γδ T cell subsets defined by Vγ usage and developmental programming (29). Murine γδ T cell functions are primarily defined in the thymus, with effector phenotypes ranging from so-called γδT1 effectors, which are characterized by CD27 and IFN-γ coexpression (30, 31), to γδT17 effectors, which lack CD27 but express CCR6 and IL17A (31, 32). Finally, mouse γδ T cells mostly reside in epithelial/mucosal compartments, encompassing a functionally unique skin-resident subset known as dendritic epidermal T cells (33–36).

**Figure 2:**
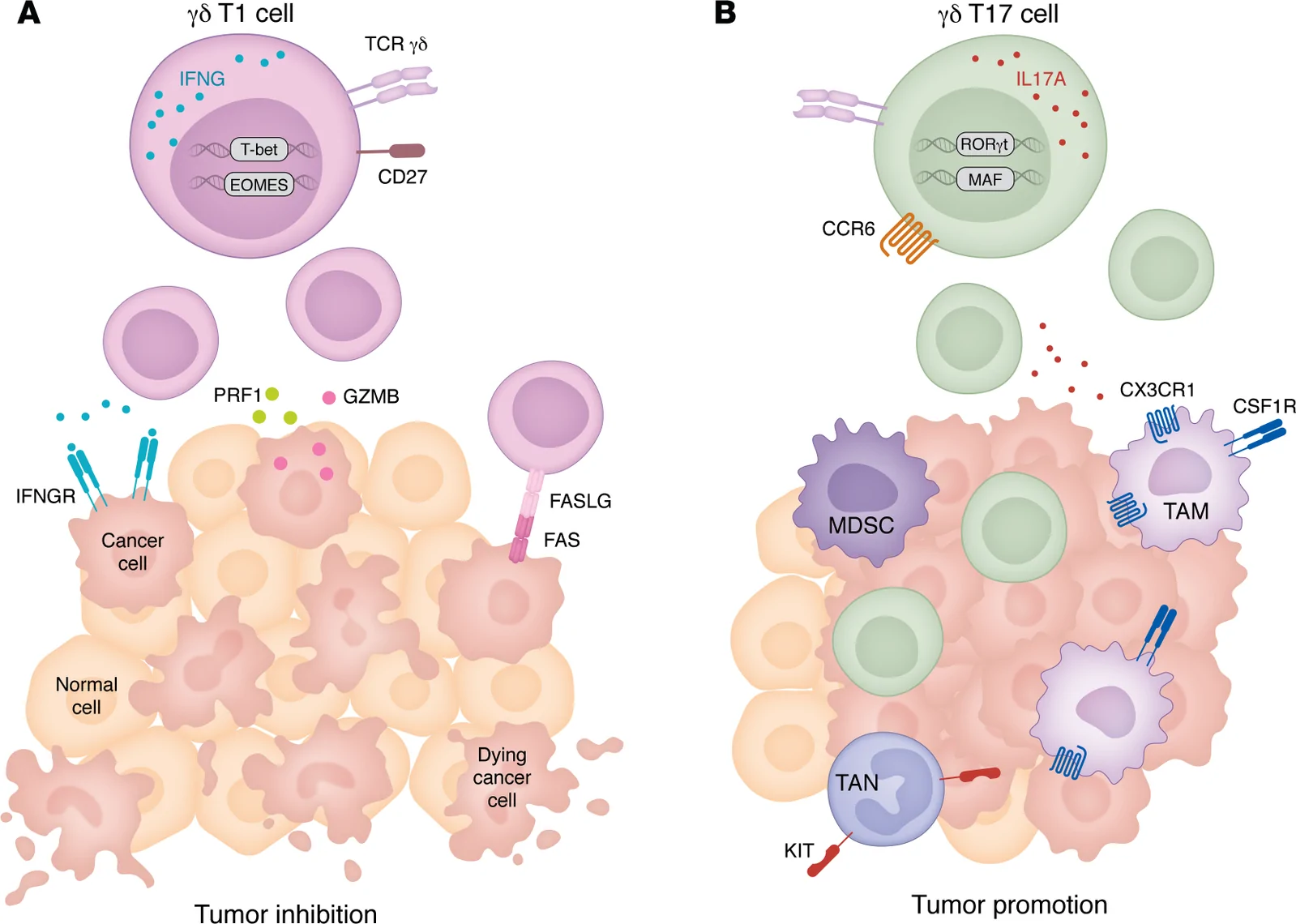
Context-dependent impact of γδ T cells on tumor progression and response to therapy. γδ T cells exist across a spectrum of phenotypic and functional phenotypes, hence impacting tumor progression and response to therapy in a highly context-dependent manner. (**A**) So-called γδT1 effectors, which require EOMES and T-bet transcription factors and are characterized by CD27 and IFN-γ coexpression, generally exert potent anticancer activity. This reflects not only the potent antineoplastic effects of IFN-γ itself, but also the ability of γδT1 cells to secrete cytotoxic effectors, including PRF1 and GZMB, and to engage death receptors on the surface of malignant cells. (**B**) Conversely, γδT17 effectors, which require MAF and RORγt transcriptional activity, lack CD27 but express CCR6 and IL17A, most often promoting cancer progression and resistance to therapy. Such an effect largely emerges from the ability of γδT17 cells to polarize the tumor microenvironment (TME) toward an immunosuppressed state dominated by CSFR1^+^ and/or CX3CR1^+^ tumor-associated macrophages (TAMs), KIT^+^ tumor-associated neutrophils (TANs), and myeloid-derived suppressor cells (MDSCs).

Accumulating evidence suggests that γδ T cells can restrain or support cancer progression depending on various intrinsic and extrinsic factors (Figure 2) (2, 37, 38). Here, we critically dissect the context-dependent interaction between γδ T cells and cancer, focusing on promising avenues underlying the manipulation of these lymphocytes for therapeutic purposes.

## γδ T cell development

γδ T cells arise through a developmental program that (a) unfolds in the thymus, (b) diverges early from αβ T cell differentiation, and (c) is shaped by TCR signaling strength and specific transcriptional networks (39–44). In both mice and humans, T cell progenitors enter the thymus as CD4^–^CD8^–^ double-negative (DN) thymocytes and progress through distinct stages (DN1–DN4 in mouse) during which the γ, δ, and β genetic loci are rearranged. Commitment to the γδ lineage is dictated by successful rearrangement and surface expression of the γδ TCR prior to DN3 and transduction of strong TCR signals, while αβ lineage commitment is associated with the formation of the so-called pre-TCR (a successfully rearranged β chain and a surrogate α chain known as pTα) and the transduction of weaker/transient signals (40, 43).

In mice, γδ T cell development occurs in ordered waves throughout embryonic and perinatal life, producing functionally distinct subsets characterized by specific TCR chain usage and homing features (29, 45). Early fetal thymic development generates Vγ5^+^ dendritic epidermal T cells, colonizing the skin, followed by Vγ6^+^ cells, which seed reproductive tissues and other epithelia (46–48). Subsequent waves produce more diverse Vγ1^+^ and Vγ4^+^ populations that circulate or reside in secondary lymphoid organs (10, 46). These waves are associated with distinct transcription factor dependencies: MAF, SOX13, and RORγt are critical for γδT17 cells (31, 32, 49–52), whereas EOMES and T-bet are required for γδT1 effectors (30, 53, 54). The transcription factor ZBTB16 (zinc finger and BTB domain containing 16) also contributes to the development of innate-like γδ T cell populations in mice, particularly those with rapid effector potential (55–58).

A key determinant of effector fate in murine γδ T cells is the strength and context of TCR signaling during thymic selection. Strong, sustained TCR signals support the differentiation of γδT1 effectors, which is associated with CD122 (IL-2 receptor β chain) upregulation and consequent sensitivity to IL-15 (42, 59). Conversely, weaker TCR signals, often along with cytokines such as TGF-β (60), favor γδT17 cell development (61, 62). NOTCH signaling also contributes to early γδ lineage decision, although less profoundly than for αβ T cell development (39).

Human γδ T cell development shares features with its mouse counterpart but lacks clearly defined developmental waves (3). Vγ9^+^Vδ2^+^ T cells are thought to arise predominantly during fetal development and expand postnatally in response to phosphorylated species (63). Conversely, Vδ1^+^ and other Vγ9^–^Vδ2^–^ γδ T cell subsets are more diverse and enriched in organs like the gut and liver, suggesting developmental programs that support tissue residency and adaptation (64).

Transcriptionally, human γδ T cell subsets segregate into functional lineages like mouse IFN-γ– and IL17A-skewed populations, although developmental timing and stability of these programs are less defined (44). Whereas T-bet and RORγt are consistently associated with IFN-γ– and IL17A-producing human γδ T cells, respectively, EOMES expression is more variable, although detectable in cytotoxic (especially Vδ1^+^) γδ T cells (65). Single-cell transcriptomic analyses also suggest that human γδ T cell development may involve branching trajectories that are influenced by thymic imprinting plus peripheral cues, including cytokines and tissue-specific signals (1).

In summary, γδ T cell development is governed by an interplay between TCR signaling and cytokine milieu, cooperatively coordinating transcriptional networks that endow differentiated γδ T cells with specific effector programs.

## Tumor-suppressive activity of γδ T cells

Since the original demonstration that γδ T cells can effectively control developing tumors (66), accumulating preclinical and clinical evidence has lent additional support to this notion. The anticancer activity of specific γδ T cell subsets (notably γδT1 effectors) reflects their capacity to (a) directly kill cancer cells (via PRF1 and GZMB or by activating FAS in target cells) or (b) release cytokines that mediate direct anti-neoplastic effects and tumor-targeting immune responses at large (e.g., IFN-γ, TNF) (38).

### γδ T cells as barriers against experimental oncogenesis.

There is abundant literature suggesting that specific γδ T cell subsets curb oncogenesis in preclinical models (Table 1). γδT1 cells effectively counteracted the progression of experimental glioblastomas in mice, a beneficial effect that was antagonized by hypoxia (67). Moreover, γδ T cells exhibiting *γδ*T1 features (e.g., IFN-γ secretion) limit disease progression in immunocompetent models of breast cancer, an effect that depends on type 1 conventional DCs and CD8^+^ cytotoxic T lymphocytes (CTLs) (68). Accordingly, orthotopically xenografted breast cancer stem cells from patients with triple-negative breast cancer (TNBC) were shown to acquire in vivo resistance to γδ T cells from healthy donors (69), supporting the existence of a selective pressure for evasion from γδ T cells in natural TNBC progression. Moreover, an intratumoral population of CD8αβ^+^ γδ T cells was shown to expand in a syngeneic mouse breast cancer model, eventually accounting for >50% of IFN-γ–producing γδ T cells, but its impact on tumor progression was not investigated (70). Of note, CD8αβ^+^ and terminally differentiated CD27^+^Ly6C^+^ γδ T cells — whose antitumorigenic abilities are established (71) — share overlapping phenotypes, but their relationship is unclear.

In mice exposed to inflammatory challenges or bearing *Apc* mutations in intestinal cells, γδ T cells colonizing the premalignant intestinal epithelium appear to restrain colorectal cancer (CRC) development, especially when they lack the transcription factor TCF-1 (72–75). However, whereas in the context of colitis-associated CRC, such protection might emerge from improved tissue homeostasis as promoted by IL-22 (72), in APC-defective intestines intraepithelial γδ T cells express IFN-γ, pointing to active immunosurveillance (73, 74). CRCs developing in *Apc^Min^* mice indeed excluded *γδ*T1 cells via a β catenin–dependent mechanism (73), culminating with relative enrichment of protumoral γδT17 effectors (74).

Specifically ablating *Ifng* from otherwise normal γδ T cells promoted the development of chemically induced sarcomas and transplantable melanomas in mice, a detrimental effect that was accompanied by limited CD8^+^ CTL reactivity against tumor lysates (76). Moreover, γδ T cells (along with CD8^+^ CTLs) have been implicated in the melanoma-suppressive effects of a junction adhesion molecule–like agonistic antibody (77) and the genetic inhibition of prostaglandin D_2_ signaling in DCs (78). Paradoxically (see below), IL17A-producing γδ T cells also appear to limit disease progression in a mouse model of gastrointestinal stromal tumor driven by KIT signaling (79).

### γδ T cells as effectors of experimental cancer therapies.

Preclinical data indicate that γδ T cells contribute to the efficacy of established and experimental anticancer regimens (Table 1). For example, the activity of recombinant IL-2 specifically targeted to CD122 against mouse bladder carcinomas has been mechanistically linked to γδ T cell activation (80). Similarly, an IL-2 variant cotargeted to CD122 and CD132 (IL-2 receptor γ chain), which avoids CD25 (IL-2 receptor α chain) signaling and consequent Treg cell–dependent immunosuppression, has been shown to expand γδ T cells in tumor-naive monkeys (81), although the functional consequences in tumor models were not assessed.

γδ T cells were required for the in vivo efficacy of intravesical Bacillus Calmette-Guérin (BCG) in both mouse and human models of bladder cancer, an effect that was maximized by pharmacological MTOR inhibition (82, 83). This aligns with ex vivo data suggesting that BCG variants can effectively expand an oligoclonal population of IFN-γ–producing CD27^+^ γδ T cells from human PMBCs (84). Intestinal γδT1 effectors have also been proposed to underlie the anticancer effects of a nanoparticle garlic formulation in melanoma-bearing mice (85), though a direct causal role for γδ T cells in the anticancer effects of this treatment was not assessed. Finally, γδ T cells expressing the cytotoxic molecule GZMA contributed to the efficacy of PD-1 and LAG3 coinhibition in a preclinical model of head and neck cancer, at least partially as effectors of anticancer immunity elicited by tumor-infiltrating FPR1^+^ neutrophils (86).

### Correlation with disease outcomes in cancer patients.

Multiple studies have linked γδ T cell infiltration/activation with improved disease outcome in patients with cancer (Table 2). The relative abundance of tumor-infiltrating Vδ1^+^ cells skewed toward IFN-γ production correlated positively with progression-free survival (PFS) and overall survival (OS) in a cohort of patients with TNBC (87), a finding that was recapitulated bioinformatically in TNBC patients from the METABRIC repository (88). Increased levels of intraepithelial Vδ1^+^ T cells expressing NKp46 in tumor-free specimens from patients with CRC were associated with a decreased risk for progression to advanced disease (89). Similarly, γδ T cell density positively correlated with extended OS in two cohorts of patients with CRC, independent of other tumor characteristics (90), and their enrichment was associated with mismatch repair (MMR) defects and (irrespective of MMR status) *B2M* (encoding beta 2 microglobulin) deficiency, which prevents MHC class I expression at the plasma membrane (90). Similar findings were obtained in an independent cohort of patients with MMR-deficient CRC (91). Reduced TCF-1 levels also correlate with γδ T cell cytotoxicity and improved survival in patients with CRC (75), lending additional support to the notion that TCF-1 limits tumor control by γδ T cells. Moreover, *TRDV1* (which encodes Vδ1) levels appear to be reduced in microsatellite-stable CRC samples compared with healthy colonic tissues, even more so in stage IV versus stage III lesions (92), corroborating the idea that intraepithelial Vδ1^+^ T cells suppress colorectal carcinogenesis.

Activated (CD69^+^) Vδ1^+^ T cell numbers in peritumoral regions correlate with limited metastatic burden and improved OS in patients with liver metastases from CRC, as do the numbers of blood-borne terminally differentiated CD69^+^Vδ1^+^ T cells (93). Whether these Vδ1^+^ T cells express Vγ9 and are activated downstream of metabolic dysregulation (94) has not been investigated. Intriguingly, human CRC appears to be infiltrated by Vδ1^+^ T cells expressing amphiregulin and exhibiting limited cytotoxicity, and expansion strategies circumventing their emergence reportedly result in γδ T cells with superior tumor-infiltrating and cytotoxic potential (37).

In patients with non–small cell lung carcinoma (NSCLC), the intratumoral abundance of CD45RA^–^CD27^–^ effector memory Vδ1^+^ T cells, as well as the numbers of tissue-resident CD103^+^Vδ1^+^ T cells in nonmalignant lung tissues, have been linked with superior PFS after surgery (95). Moreover, a genetic signature of Vδ2^+^ T cells expressing NKG2A reportedly correlates with improved OS in patients with NSCLC and hepatocellular carcinoma (HCC) (96). The positive impact of tissue-resident γδ T cells on survival in patients with HCC has been validated in an independent study, irrespective of the relative depletion of Vγ9^+^Vδ2^+^ T cells (97). In contrast, the prognostic impact of Vδ2^+^ T cells in glioblastoma remains to be clarified. Indeed, intratumoral Vδ2^+^ T cells have been reported to positively correlate with disease outcomes in one study (98), but in an independent work, Vδ2^+^ T cell signatures failed to correlate with improved OS, possibly because of overexpression of the immunosuppressive NKG2A ligand HLA-E (96).

The intratumoral abundance of γδ T cells, specific subsets thereof, and/or their ex vivo sensitivity to stimulation have also been associated with improved outcome in cohorts of patients with bladder (99), cervical (100), renal (101), and ovarian (102) cancer, but not medulloblastoma, despite pronounced variations across patient subgroups (103). In ovarian cancer, Treg cells were inferred to be responsible for the contraction of γδ T cell responses associated with metastatic dissemination and poor outcome (104), potentially downstream of IL-2 depletion (105). Moreover, in patients with cervical cancer, genetic signatures of γδ T cell exhaustion correlated with BTN3A1 expression (as part of a negative feedback loop elicited by IFN-γ) and poor disease outcome (106), reinforcing the notion that cervical cancer is under immunosurveillance by IFN-γ–producing γδ T cells. Finally, the intratumoral levels of *TRDC* (a proxy for γδ T cell abundance) were associated with superior OS in patients with resected gastrointestinal stromal tumor (79).

### Correlation with therapeutic responses in cancer patients.

γδ T cell infiltrates have been correlated with improved sensitivity to treatment in patients affected by various neoplasms (Table 2). The γδ T cell fraction, as assessed by immune-related metagenes at baseline, was associated with PCR in patients with HER2^+^ breast cancer receiving neoadjuvant therapy (107). Similarly, inflammatory breast cancer (which is sensitive to immune checkpoint inhibitors [ICIs]) (108, 109) appears to exhibit a superior γδ T cell infiltrate compared with its noninflammatory counterpart (110), and patients with TNBC responding to a PD-L1 blocker manifested a longitudinal increase in circulating γδ T cells compared with nonresponders (111).

In patients with hepatic metastases from MMR-defective CRC, circulating IL-18 levels have been associated with improved sensitivity to ICIs through preserved IFN-γ secretion by intratumoral Vδ1^+^ T cells (112). Moreover, intratumoral Vδ1^+^ and Vδ3^+^ T cell levels were increased after treatment with ICIs specific for CTLA4 and PD-1 in an independent cohort of patients with MMR-deficient CRC lacking B2M (91). Transcriptional programs linked to retained effector functions in Vδ2^–^ T cells were also enriched in patients with renal cell carcinoma (RCC) responding to a PD-L1 blocker versus nonresponders (101). Furthermore, the intratumoral levels of *TRDV1* and the abundance of tumor-infiltrating Vδ1^+^ T cells appear to predict responsiveness to PD-1 inhibitors in patients with melanoma (113, 114), perhaps even more profoundly when intratumoral Vδ1^+^ T cells downregulate inhibitory KIR receptors during treatment (115). A similar connection between intratumoral Vδ1^+^ T cells and sensitivity to ICIs has been documented in patients with Merkel cell carcinoma (116, 117). That said, γδ T cells from sentinel lymph nodes of melanoma patients express increased CTLA4 levels (118), potentially indicating exhausted or otherwise dysfunctional phenotypes (119).

Thus, γδ T cells not only mediate anticancer effects in preclinical tumor models, but also correlate with improved cancer outcomes in clinical settings. More specifically, human Vδ1^+^ T cells stand out as a powerful tumor-targeting γδ T cell subset, although some Vδ2^+^ populations also exhibit antineoplastic activity. Numerous strategies harnessing these γδ T cell populations for therapeutic purposes are being developed.

## Tumor-promoting activity of γδ T cells

Specific γδ T cell populations promote tumor progression, often by favoring the establishment of an immunosuppressive tumor microenvironment (TME). Such an activity may result not just from the reconfiguration of intratumoral cytokine networks as driven by IL17A, TGF-β, and IL-10, but also from the contact-dependent inhibition of immune effectors (120).

### γδ T cells as drivers of experimental oncogenesis.

An expanding preclinical literature suggests that specific γδ T cell subsets promote tumor progression (Table 3). IL17A-producing γδ T cells have been associated with accrued metastatic dissemination in multiple models of TNBC (121, 122), in some cases by promoting the expansion of immature neutrophils expressing KIT that precondition metastatic niches (122). In line with these findings, neoplastic lesions relapsing after the genetic eradication of rapidly proliferating malignant cells in mice expressing the polyomavirus middle T antigen in mammary epithelia were enriched in γδ T cells expressing IL17A and RORγt (123). Intriguingly, the tumor-promoting activity of IL17A-secreting γδ T cells may be related to the expression of the coinhibitory receptor TIM-3 by some breast cancer cell populations (121), pointing to TIM-3 as a promising target to intercept the expansion of tumor-promoting γδ T cells prior to TNBC dissemination. Moreover, γδT17 effectors appear to expand (intratumorally and systemically) in mice bearing syngeneic TNBC lesions and exposed to a high-fat diet, at least partially owing to the ability of γδT17 cells to efficiently metabolize lipids (124). However, whether the tumor-promoting effects of a high-fat diet are mechanistically linked to IL17A-secreting γδ T cells remains to be established.

Tumor promotion by IL17A-producing γδ T cells and KIT^+^ neutrophils has also been documented in mouse models of anal cancer based on KRAS^G12D^ expression in transition zone epithelial cells and repeated wounding (125). Similarly, RORγt-dependent IL17A-producing Vγ4^+^ and Vγ6^+^ cells have been mechanistically implicated in colitis-driven CRC in mice (74). Moreover, γδ T cell subsets expressing CD39, an ectonucleotidase with prominent immunosuppressive effects (126, 127), appear to promote local and distant tumor progression in preclinical models of right-sided CRC, at least partially resulting from differential arachidonic acid metabolism in right-sided versus left-sided CRC cells (128). A similar tumor-promoting activity has been attributed to γδ T cells in preclinical models of NSCLC (129) and pancreatic ductal adenocarcinoma (PDAC) (130). In NSCLC-bearing mice, MAF (a critical specifier of the γδT17 effector fate) (50) was required for γδ T cells to accelerate disease progression (129), highlighting it as a potential target to inhibit tumor-supporting γδ T cells. Finally, a population of CD27^–^Vγ1^+^ T cells has been proposed to promote a profibrotic program in pulmonary macrophages (131). While this program was associated with poor outcome in patients with lung adenocarcinoma, however manipulating Vγ1^+^ T cells had no impact on tumor progression in preclinical NSCLC models (131).

### γδ T cells as barriers against experimental cancer therapies.

Emerging preclinical evidence suggests that specific γδ T cell populations limit the efficacy of common anticancer therapeutics (Table 3). For instance, IL17A-producing γδ T cells were shown to counteract the (immuno)therapeutic effects of CDK4/6 inhibitors (132) in a mouse model of HR^+^HER2^–^ breast cancer, at least partially by promoting the repolarization of tumor-associated macrophages (TAMs) toward an immunosuppressive phenotype (133, 134). Along similar lines, Vγ4^+^ and Vγ6^+^ T cells secreting IL17A mediated resistance to ICIs targeting TIM-3 and PD-1 in preclinical models of breast cancer, largely reflecting the ability of TIM-3 and PD-1 blockers to promote the expansion of these immunosuppressive γδ T cell subsets (135), Moreover, γδ T cells were decreased in the microenvironment of experimental TNBC effectively controlled by chemotherapy or ICIs (136).

IL17A-producing Vγ1^+^ T cells have been associated with resistance to ICIs in preclinical models of CRC and B cell lymphoma, reflecting their ability to repolarize the TME toward an immunosuppressive configuration involving myeloid-derived suppressor cells (MDSCs) upon activation by BTNL2^+^ malignant cells (137). Likewise, IL17A-secreting IL23R^+^ γδ T cells reportedly limit the therapeutic efficacy of radiation therapy in immunocompetent mice bearing subcutaneous NSCLCs (138). Interestingly, in this latter setting, γδ T cell recruitment was ascribed to CCL20 secretion by a population of TAMs engulfing tumor-derived microparticles containing dsDNA and hence activating indolent STING signaling (138). This is aligned with an expanding literature indicating that indolent STING responses paradoxically favor the establishment of an immunosuppressive TME (139).

### Negative correlation with disease outcomes in cancer patients.

The ability of specific populations of γδ T cells to support tumor progression has also been documented in clinical datasets (Table 4). Intratumoral γδ T cells positively correlated with tumor stage, HER2 expression, and lymph node involvement, as well as shortened survival, in a cohort of patients with multiple breast cancer subtypes (140). Likewise, in patients with HR^+^HER2^–^ breast cancer, intratumoral γδ T cell abundance correlated positively with disease stage (133). Moreover, genetic signatures of γδ T cells or active IL17A signaling were associated with shortened OS in patients with HR^+^HER2^–^ breast cancer from public transcriptomic repositories (133). Finally, circulating Vδ1^+^ T cells from patients with metastatic TNBC appear to produce increased IL17A levels compared with Vδ1^+^ T cells from healthy women, an alteration that was associated with functional shifts in circulating neutrophils (141).

### Negative correlation with therapeutic responses in cancer patients.

A higher-than-median circulating count for activated (IL7R^+^) γδ T cells at baseline in patients with HR^+^HER2^–^ breast cancer has been associated with shortened PFS upon treatment with CDK4/6 inhibitors and endocrine therapy (133). Similarly, in a small set of paired biopsies from women with HR^+^HER2^–^ breast cancer undergoing the same treatment, intratumoral γδ T cells were markedly increased at disease relapse (133). Moreover, the circulating levels of Vδ1^+^ T cells at baseline have been associated with reduced OS in patients with melanoma receiving ICIs, potentially reflecting a senescent-like phenotype that was not evident in intratumoral Vδ1^+^ T cells (114). Finally, γδ T cells manifesting transcriptional signatures of cell cycle progression and suppressed immune signaling have been associated with resistance to ICIs in a cohort of patients with melanoma (142).

Altogether, these observations suggest that specific γδ T cell subsets promote tumor progression and resistance to (immuno)therapy. In this context, γδT17 effectors stand out as promising targets for the development of novel therapeutic strategies against a variety of neoplasms.

## Harnessing γδ T cells for cancer therapy

**Figure 3:**
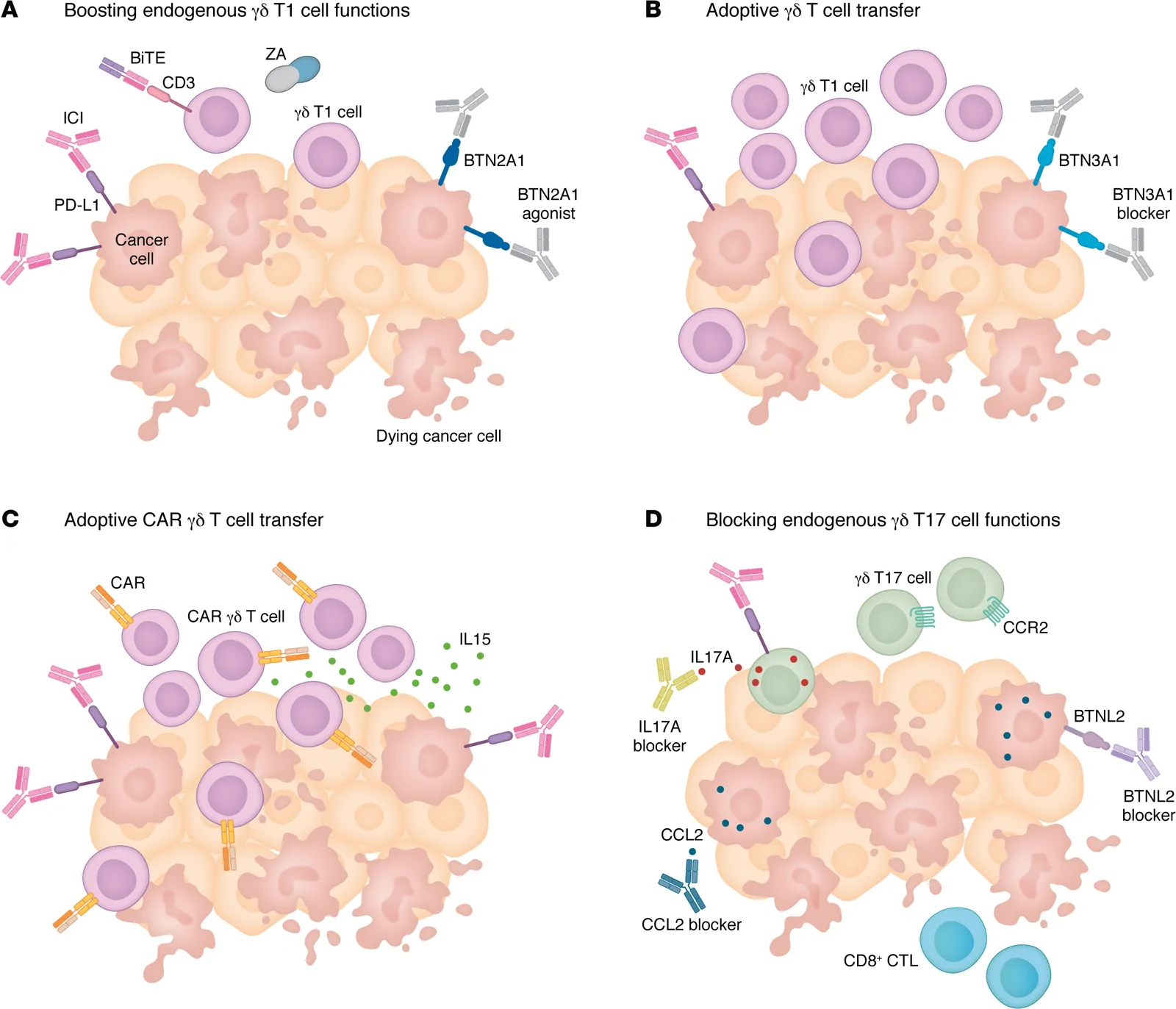
Therapeutic strategies harnessing γδ T cells for cancer therapy. Multiple strategies harnessing the immunobiology of γδ T cells are being explored to expand our therapeutic armamentarium against malignant disorders. Such strategies encompass approaches that leverage the potent anticancer effects of γδT1 cells, such as (**A**) the administration of agents reinvigorating or engaging endogenous γδT1 cells, including ZA, BTN2A1 agonists, γδ T cell–targeting BiTEs, and ICIs; (**B**) the infusion of ex vivo expanded (but otherwise unmodified) autologous or allogenic γδ T cells, optionally along with immunostimulatory agents like ICIs or BTN3A1 blockers; (**C**) the infusion of ex vivo expanded γδ T cells that have been engineered to express a tumor-targeting CAR and/or immunostimulatory molecules (e.g., IL-15), also optionally in combination with ICIs or other immunostimulants. (**D**) Blocking the immunosuppressive activity of γδT17 cells has also been shown to mediate anticancer effects (at least in preclinical tumor models). The latter paradigm has largely been built on strategies interfering with γδT17 cell recruitment, including focal radiation therapy as well as BTNL2 and CCL2 blockage, or functions, including PD-L1 or IL17A blockage, ultimately in support of restored CD8^+^ CTL responses.

Emerging evidence indicates that γδ T cells can effectively be exploited for cancer therapy, based on two major paradigms: (a) harnessing and/or boosting the natural propensity of IFN-γ–producing γδ T cells to mediate anticancer effects and (b) inhibiting the tumor-promoting activity of IL17A-secreting γδ T cells (Figure 3).

### Activating endogenous γδ T cells.

Engaging endogenous γδT1 cells has been shown to effectively control disease progression in preclinical tumor models. For instance, antibody-drug conjugates specific for EGFR and loaded with zoledronic acid (ZA) analogs exerted therapeutic activity linked to Vδ2^+^ T cell expansion in preclinical CRC models (143). Likewise, ZA, BCG, and a BTN3-specific agonistic antibody elicited robust activation of Vδ2^+^ T cells isolated from human bladder neoplasms (99). A BTN2A1 agonist has also been shown to promote the antineoplastic effects of Vγ9^+^Vδ2^+^ T cells against various human cancer cell lines and primary acute lymphoblastic leukemia cells (144). Such an effect may also be achievable with pharmacological AMPK activators, reflecting the ability of AMPK to control BTN2A1 and BTN3A expression in malignant cells (145).

A CD34- and CD3-targeting bispecific T cell engager (BiTE) has been shown to effectively engage γδ T cells against acute myeloid leukemia targets while sparing healthy CD34^+^ hematopoietic stem cells as well as CD34^dim^ endothelial cells from the blood-brain barrier (146). Similar efficacy was documented with a BiTE specific for Vγ9^+^Vδ2^+^ T cells and CD19 in preclinical models of CD19^+^ lymphoma (147), although in vivo toxicity against nonmalignant CD19^+^ cells was not assessed. A Vδ2- and PD-L1–specific BiTE has recently been reported to trigger Vδ2^+^ T cell cytotoxicity against human melanoma and RCC cellular systems in vitro (148). Interestingly, this activity was associated with the Vδ2^+^ T cell–dependent elimination of PD-L1^+^ myeloid cells as well as the upregulation of activation markers in CD4^+^ and CD8^+^ T cells (148).

### γδ T cell–based cell therapies.

The adoptive transfer of natural or genetically engineered γδ T cells has also been consistently associated with anticancer activity in preclinical tumor models, a beneficial effect that in many cases could be boosted by expanding γδ T cells in specific culture conditions of coadministering pharmacological agents limiting dysfunction. Adoptively transferring human Vγ9^+^Vδ2^+^ cells effectively controlled human TNBC lesions developing in immunodeficient mice, even more so when delivered along with the HIF1A inhibitor PX478 (149). A similar therapeutic effect has been documented in immunocompetent murine models of breast cancer upon adoptive transfer of mouse γδ T cells exhibiting features of γδT1 effectors (e.g., CD27 expression, IFN-γ secretion), especially when expanded ex vivo with high-dose glucose (71, 124). Moreover, human γδ T cells exerted robust antineoplastic activity against leukemia, both in vitro and in vivo, a therapeutic effect that was more pronounced when γδ T cells were isolated after physical exercise (potentially owing to increased adrenergic signaling) (150).

Human Vδ1^+^ cells expanded in vitro (also known as Delta One T or DOT cells) (38, 151) efficiently killed human CRC cell lines and organoids, both in vitro and in vivo (in immunodeficient mice), by a mechanism that can be boosted by butyrate (increasing the expression of NKG2D ligands on neoplastic cells) as well as dual PD-1 and TIGIT blockage (152). A similar effect has been documented in mice bearing human NSCLC xenografts and inoculated with Vδ1-enriched γδ T cells in the optional presence of DNA methyltransferase inhibitors, which increased efficacy by upregulating γδ T cell ligands on malignant cells (153). An ICI specific for PD-1 alone or combined with other immunostimulatory agents also efficiently improved the therapeutic activity of adoptively transferred human Vγ9^+^Vδ2^+^ cells in immunodeficient mice bearing human solid tumor xenografts (154, 155).

Blocking BTN3A1 (which is expressed by malignant cells responding to IFN-γ and promotes γδ T cell exhaustion) (106) also appears to enhance the activation of adoptively delivered Vγ9^+^Vδ2^+^ T cells and restore (adoptively transferred) αβ T cell activation in immunodeficient mice bearing human ovarian carcinoma (156). Additional strategies that have been successfully employed with this objective include providing γδ T cells with TGF-β during expansion (157) as well as coadministering them with retinoic acid, a strategy that robustly limited TIM-3 expression by γδ T cells (and dysfunction at large) in preclinical models of nasopharyngeal carcinoma (158).

Promising observations have also been made with γδ T cells genetically engineered to secrete synthetic tumor-targeting antibodies along with an IL15-IL15RA fusion protein (159) or artificially redirected against EGFR-expressing cancer cells upon conjugation with an EGFR-specific antibody (160). That said, recent efforts aimed at the generation of powerful γδ T effectors for adoptive cell transfer have focused on the CAR technology, building on the expectation that γδ T cells may be better suited than αβ T cells for an off-the-shelf CAR T product (161). The therapeutic potential of adoptively transferred CAR γδ T cells has been documented in several human xenograft models established in immunodeficient mice, including models of (a) acute myeloid leukemia, based on DOT cells targeting IL3RA (162); (b) HIV-1–associated B cell lymphoma, harnessing γδ T cells targeting CD19 and rendered CCR5 defective (hence, HIV-1 resistant) (163); (c) HCC, using DOT cells targeting GPC3 and engineered to secrete IL-15 (164); (d) NSCLC, based on γδ T cells targeting EGFR and engineered to coexpress CCR6 and a soluble PD-1 blocker (165); (e) PDAC, harnessing DOT cells or Vδ2^+^ T cells targeting PSCA (166); (f) ovarian cancer, using DOT cells targeting ERBB2 (167); (g) prostate carcinoma, with Vγ9^+^Vδ2^+^ CAR T cells targeting PSCA and expanded with ZA (168); and (h) various other hematological and solid neoplasms, based on γδ T cells targeting CD70 and expressing dominant-negative TGF-β receptors (169).

Lending support to durable anticancer activity of γδ T cells, a population of CAR γδ T cells has been shown to persist in the circulation of patients with chronic lymphocytic leukemia more than a decade after complete remission upon receiving CD19-redirected CAR T cells (170). Moreover, ZA-expanded autologous Vγ9^+^Vδ2^+^ T cells were well tolerated and associated with disease control rate of 68% in 25 patients with NSCLC enrolled in a single-arm, phase II clinical trial, although the primary objective of the study (PFS > 4 months) was not met (171). Similar findings have been documented in preliminary form in several other clinical trials testing γδ T cells in patients with cancer (172–174), but clinical data remain immature at this stage (92, 175, 176).

### Preventing γδ T cell dysfunction or tumor promotion.

An alternative therapeutic strategy is to intercept (or actively reverse) γδ T dysfunction in progressing neoplasms. This can be achieved with ICIs, including conventional PD-1 inhibitors, which effectively restored tumor-targeting γδ T cell responses against cultured human breast cancer stem-like cells (175). In addition, investigational agents, like TIGIT blockers, have been effectively harnessed to recover the cytotoxic activity of Vδ1^+^ T cells against human CRC cells in vitro (92). A similar improvement in the cytotoxic activity of γδ T cells against cultured human breast cancer stem-like cells was achieved with the metalloprotease inhibitor GW280264X, at least partially reflecting suppressed MICA shedding from malignant cells (175). Moreover, γδ T cell activation against human neuroblastoma cells elicited by ganglioside D2 (GD2)-specific antibodies can be effectively boosted by suppressing YAP signaling, largely as a consequence of increased GD2 synthesis (176).

Effectively inhibiting the tumor-promoting activity of specific γδ T cell populations has also been associated with antineoplastic effects in preclinical tumor models. For instance, PDAC-promoting γδ T cells were shown to express elevated levels of PD-L1, and blocking it with a specific ICI effectively restored tumor control by αβ T cells in a mouse model of this aggressive tumor (130). Similarly, IL17A-secreting γδ T cells have been shown to promote the resistance of HR^+^HER2^–^ mammary tumors to CDK4/6 inhibitors plus endocrine therapy, and interrupting their recruitment (with radiation therapy or a CCL2 neutralizer) or activity (with a γδ TCR blocker or an IL17A neutralizer) has been associated with superior treatment sensitivity in mice (133, 177, 178). Of note, IL17A-secreting γδ T cells appear to be recruited to various solid malignancies via a BTNL2-dependent mechanism, and in mice, interrupting this pathway effectively restores immunological tumor control (137).

### Additional strategies.

Promising but hitherto underdeveloped therapeutic approaches based on the γδ T cell biology include (a) bispecific molecules involving a CD3-targeting moiety and the extracellular domains of tumor-reactive Vγ9Vδ2 TCRs to effectively redirect αβ T cells to cancer cells (179), (b) the delivery of γδ T cell–derived exosomes as an approach to improve radiosensitivity (180), and (c) the use of γδ TCR sequences to engineer CARs for transducing conventional, unselected for lineage, T cells (181, 182).

## Conclusions and outlook

Multiple treatments that build on the immunobiology of γδ T cells and cancer are emerging, including strategies under active clinical development (Table 5). However, there are numerous obstacles to unlocking the full potential of γδ T cells as actionable targets for cancer immunotherapy.

First, unlike conventional αβ T cells, γδ T cells often fail to sustain proliferation for long periods after infusion, reducing the durability of responses. Strategies that are being investigated to circumvent this issue include the transgene-enforced or pharmacological provision of mitogenic cytokines (e.g., IL-2, IL-15) and/or activating molecules (e.g*.*, ICIs) (157, 159, 164, 165, 183–186), the development of improved ex vivo expansion protocols (167, 187), and repeated dosing (184).

Second, different γδ T cell subsets, even when cytotoxic a priori (e.g., Vδ1^+^ vs. Vγ9^+^Vδ2^+^), exhibit considerably divergent functions, which complicates clinical applicability, especially in the context of immunosuppressive TMEs (37, 95, 101, 188). A deeper understanding of the (immuno)biology of specific γδ T cell populations, enabling selective expansion protocols and genetic engineering strategies to fine-tune anticancer activity, is required to overcome this limitation.

Third, factors such as TGF-β, hypoxia, and abundant levels of MDSCs and/or immunosuppressive TAMs impair the antineoplastic effects of γδ T cells (67, 189–191). In this context, combinatorial regimens involving ICIs, metabolic reprogrammers, and/or additional agents that remodel the TME represent promising avenues to restore the anticancer activity of natural or adoptively transferred γδ T cells.

Finally, selective targeting strategies are lacking, especially for approaches aiming to inhibit or eliminate tumor-promoting γδ T cells, as interfering with cytokine signaling at large may impair healthy tissue homeostasis (e.g., in epithelial linings) (192–195), highlighting an urgent need for the development of more specific interventions.

In conclusion, γδ T cells represent a promising, though still evolving, platform for cancer therapy. Addressing these and other obstacles may soon lead to the development of clinically approved therapeutic protocols building on this peculiar lymphocyte subset.

## Author contributions

LB and LG conceived the article. LB and LG wrote the first version of the manuscript with critical input from SBC, BSS, and DLW. LB prepared display items under supervision from LG. All authors approved the submitted version of the article.

## Conflict of interest

BSS holds a research contract with Takeda Development Center Americas. LG holds or has held research contracts with Lytix Biopharma, Promontory, and Onxeo; has received consulting/advisory honoraria from Boehringer Ingelheim, AstraZeneca, OmniSEQ, Onxeo, The Longevity Labs, Inzen, Imvax, Sotio, Promontory, Noxopharm, EduCom, and the Luke Heller TECPR2 Foundation; and holds Promontory stock options.

## Funding support

This work is the result of NIH funding, in whole or in part, and is subject to the NIH Public Access Policy. Through acceptance of this federal funding, the NIH has been given a right to make the work publicly available in PubMed Central.

- Kirby Training Fellowship from Fox Chase Cancer Center (to LB).
- Breast Cancer Now (2024.11PR1736, 2019DecPhD1349, and 2018JulPR1101 to SBC).
- Cancer Research Institute (CRI3692 to SBC).
- Cancer Research UK (A25142, CTRQQR-2021\100006, DRCNPG-Jun22\100007, PRCBRP-Nov23/100019, and RCCCEA-Nov21\100003 to SBC).
- Annie McNab Bequest (to the Cancer Research UK Scotland Institute).
- Medical Research Council (MR/X018326/1 to SBC).
- Medical Research Scotland (PHD-50669-2023 to SBC).
- Wellcome Trust (208990/Z/17/Z to SBC).
- Worldwide Cancer Research/Pancreatic Cancer UK (22-00135 to SBC).
- Research agreement with Takeda Development Center Americas (BSS).
- NIH grant P01AI102853 (to DLW).
- NIH R01 grant (CA271915 to LG).
- Breakthrough Level 2 grants from the US Department of Defense Breast Cancer Research Program (BC180476P1 and BC210945 to LG’s lab).
- STARR Cancer Consortium grant (I16-0064 to LG).
- Transformative Breast Cancer Consortium Grant from the US Department of Defense Breast Cancer Research Program (W81XWH2120034 to Silvia C. Formenti).
- U54 grant from NIH/National Cancer Institute (CA274291 to Joseph O Deasy, Silvia C Formenti, and Ralph Weichselbaum).
- 2019 Laura Ziskin Prize in Translational Research from Stand Up to Cancer (ZP-6177 to Silvia C Formenti).
- Mantle Cell Lymphoma Research Initiative grant from the Leukemia and Lymphoma Society (to Selina Chen-Kiang).
- Rapid Response Grant from the Functional Genomics Initiative (to LG).
- Pre-SPORE grant (to Sandra Demaria and Silvia C Formenti).
- Collaborative Research Initiative Grant and Clinical Trials Innovation Grant from the Sandra and Edward Meyer Cancer Center (to LG).
- Startup funds from the Department of Radiation Oncology at Weill Cornell Medicine (to LG).
- Startup funds from Fox Chase Cancer Center (to LG’s lab).
- Industrial collaborations with Lytix Biopharma, Promontory, and Onxeo (to LG).
- Donations from Promontory, the Luke Heller TECPR2 Foundation, Sotio a.s., Lytix Biopharma, Onxeo, Ricerchiamo, Noxopharm, The Eileen Stein Jacoby Fund, Dora Vardaro, and Pat Pasquarello (to LG).

## Figures and Tables

**Table 5:**
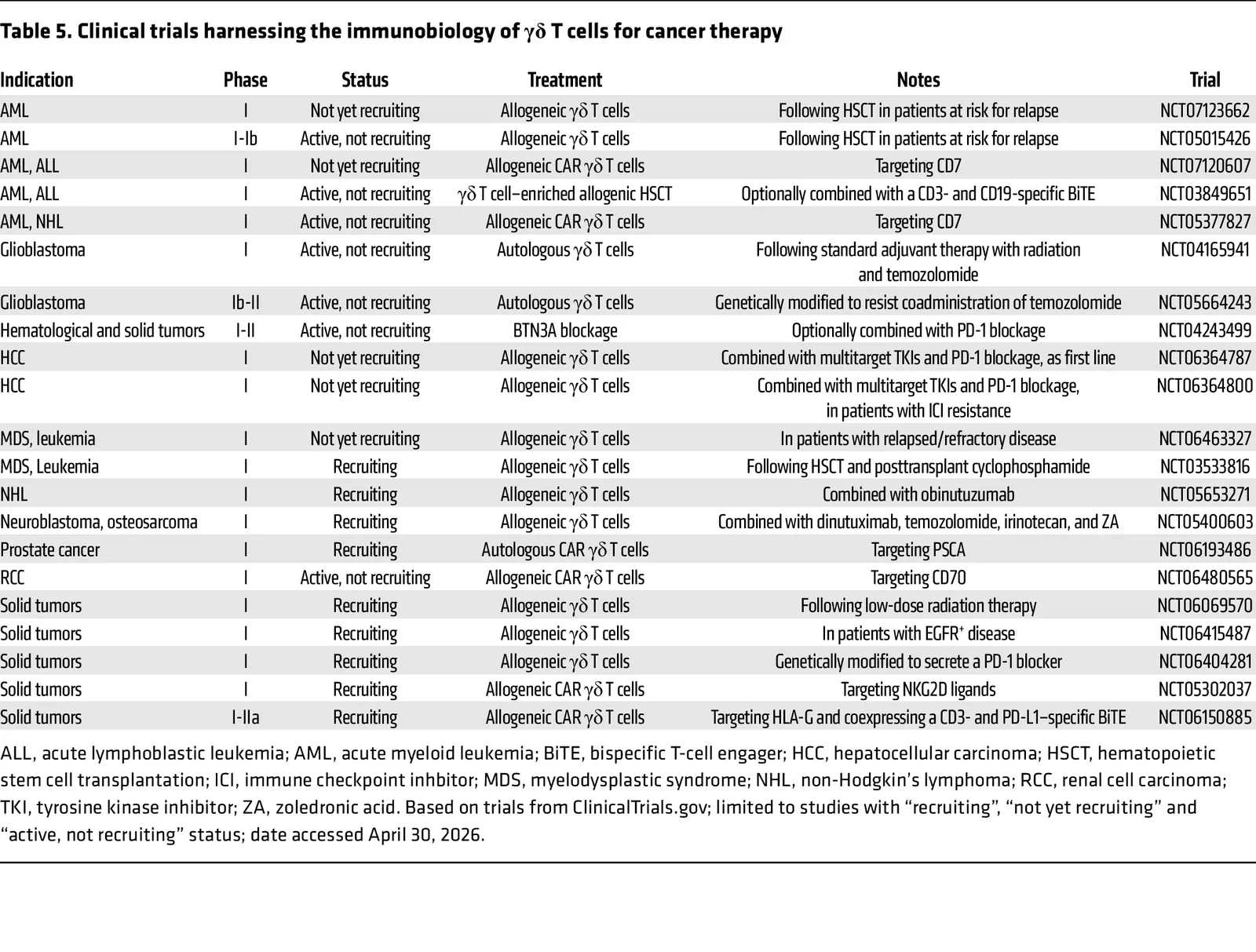
Clinical trials harnessing the immunobiology of γδ T cells for cancer therapy

**Table 4:**
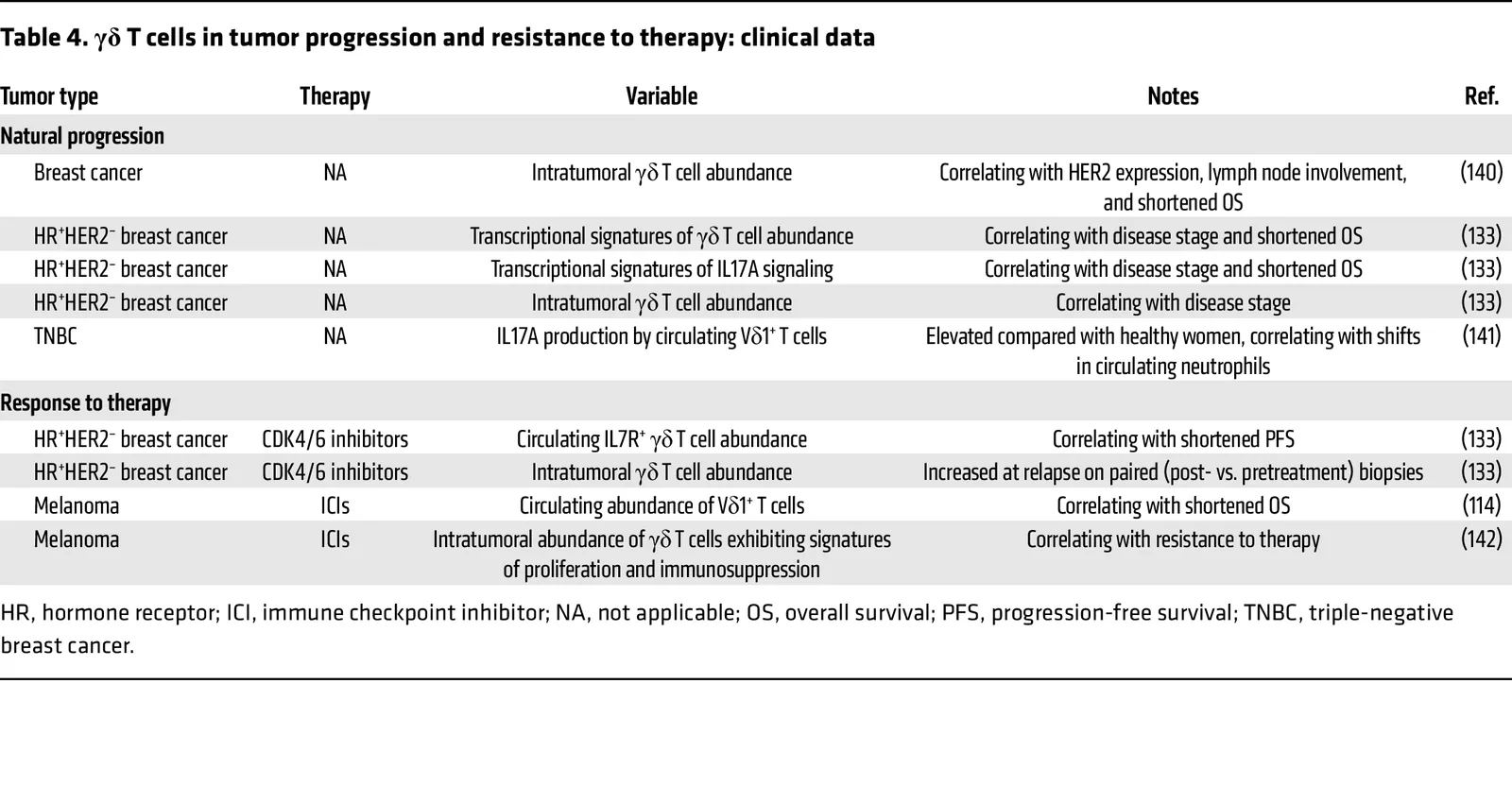
γδ T cells in tumor progression and resistance to therapy: clinical data

**Table 3:**
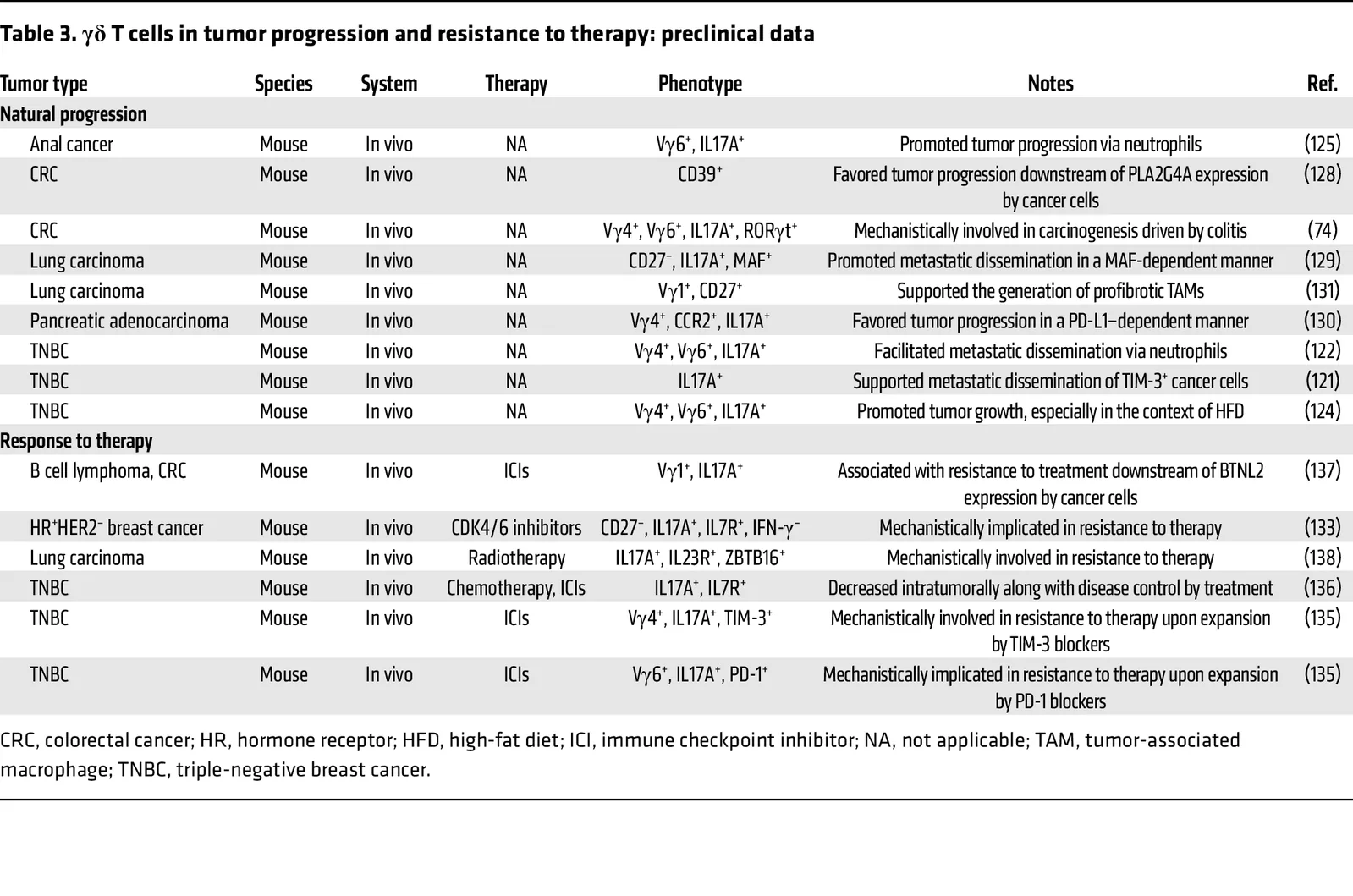
γδ T cells in tumor progression and resistance to therapy: preclinical data

**Table 2:**
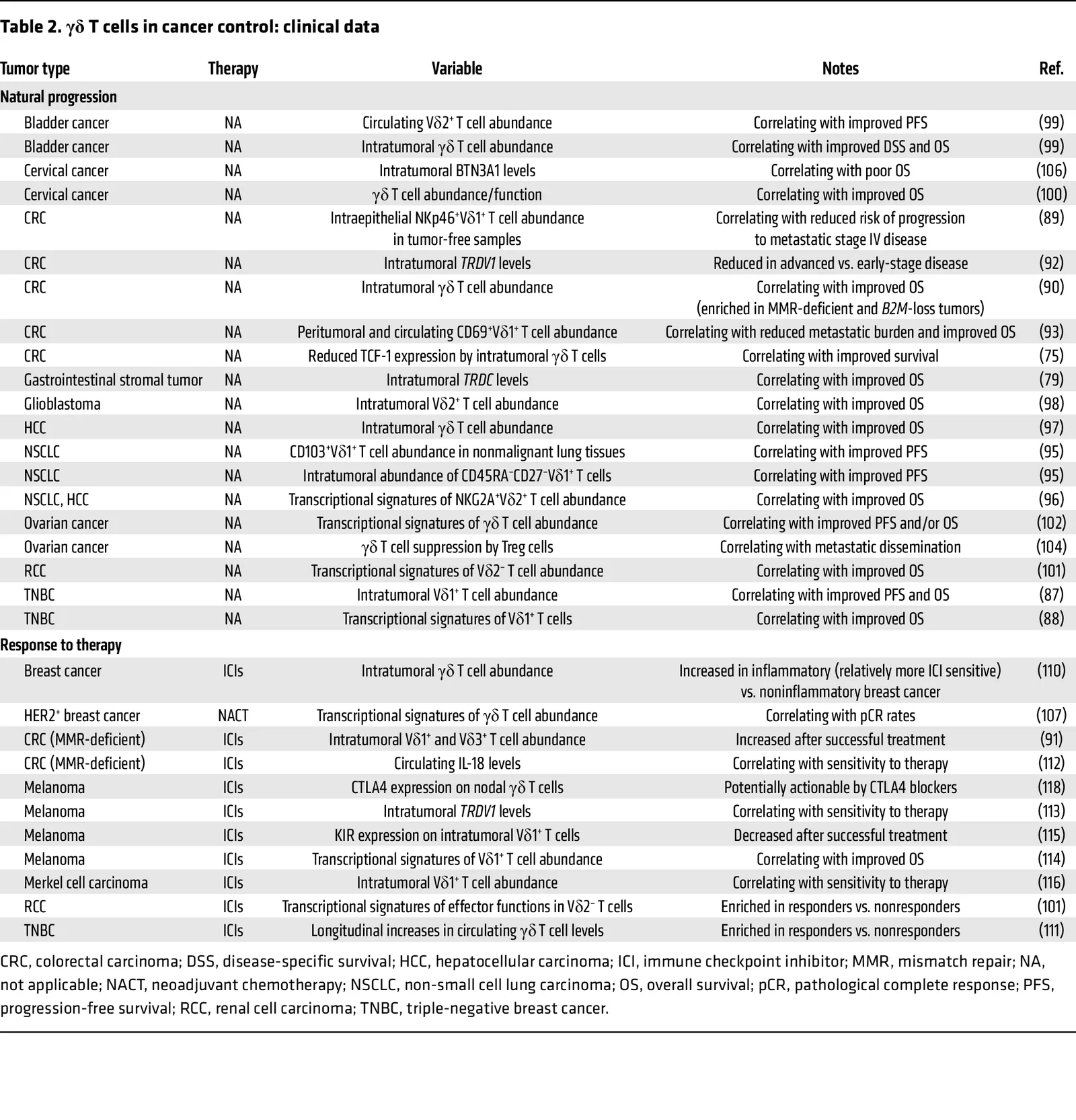
γδ T cells in cancer control: clinical data

**Table 1:**
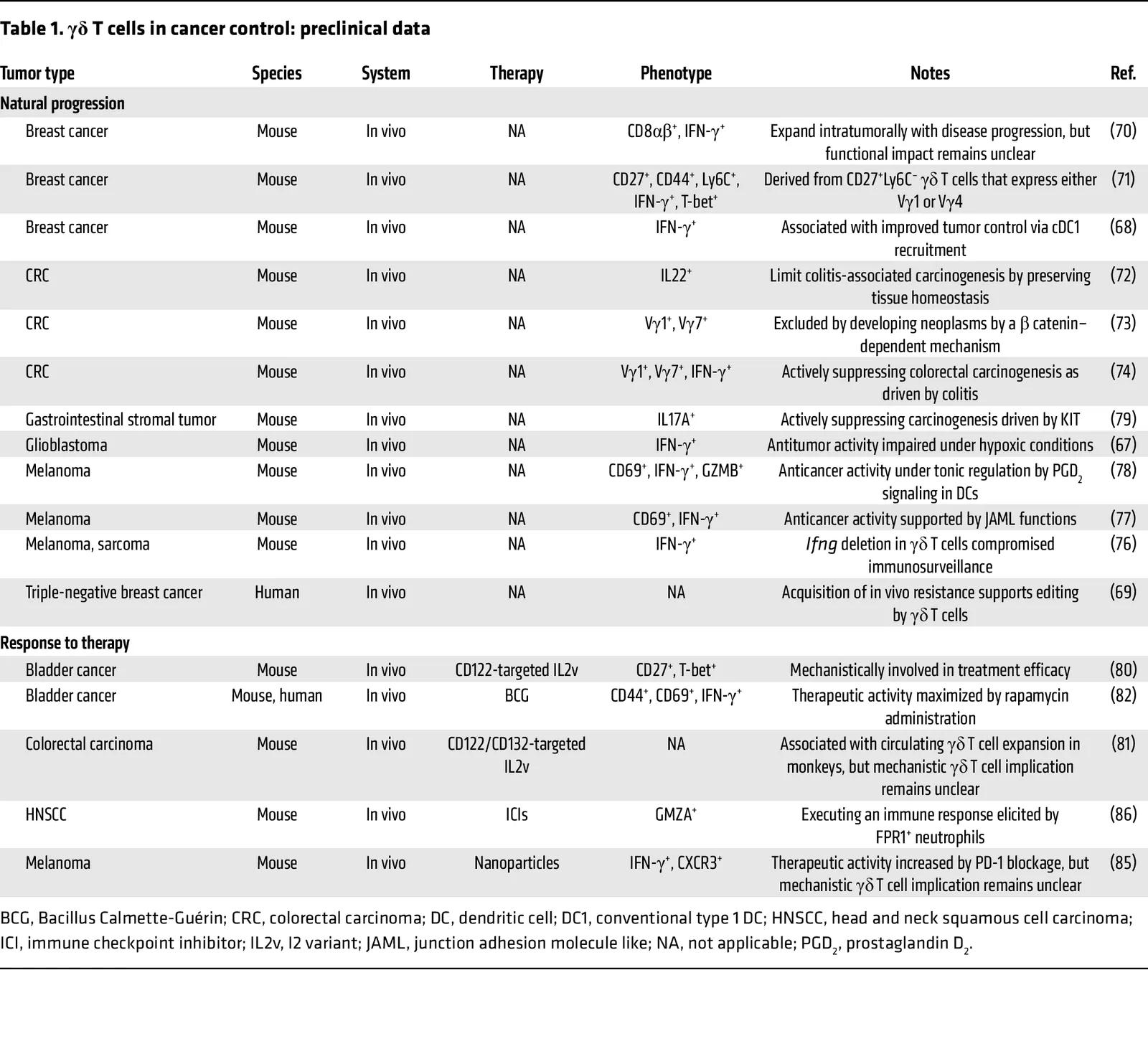
γδ T cells in cancer control: preclinical data

## Version 1 09/01/2026

Electronic publication
